# Modeling Fence Location and Density at a Regional Scale for Use in Wildlife Management

**DOI:** 10.1371/journal.pone.0083912

**Published:** 2014-01-08

**Authors:** Erin E. Poor, Andrew Jakes, Colby Loucks, Mike Suitor

**Affiliations:** 1 Department of Fish and Wildlife Conservation, Virginia Tech, Blacksburg, Virginia, United States of America; 2 Conservation Science Program, World Wildlife Fund, Washington D.C., United States of America; 3 Faculty of Environmental Design, University of Calgary, Calgary, Canada; 4 Environment Yukon, Dawson City, Yukon, Canada; University of Georgia, United States of America

## Abstract

Barbed and woven wire fences, common structures across western North America, act as impediments to wildlife movements. In particular, fencing influences pronghorn (*Antilocapra americana*) daily and seasonal movements, as well as modifying habitat selection. Because of fencing's impacts to pronghorn and other wildlife, it is a potentially important factor in both wildlife movement and habitat selection models. At this time, no geospatial fencing data is available at regional scales. Consequently, we constructed a regional fence model using a series of land tenure assumptions for the Hi-Line region of northern Montana – an area consisting of 13 counties over 103,400 km^2^. Randomized 3.2 km long transects (n = 738) on both paved and unpaved roads were driven to collect information on habitat, fence densities and fence type. Using GIS, we constructed a fence location and a density model incorporating ownership, size, neighboring parcels, township boundaries and roads. Local knowledge of land ownership and land use assisted in improving the final models. We predict there is greater than 263,300 km of fencing in the Hi-Line region, with a maximum density of 6.8 km of fencing per km^2^ and mean density of 2.4 km of fencing per km^2^. Using field data to assess model accuracy, Cohen's Kappa was measured at 0.40. On-the-ground fence modification or removal could be prioritized by identifying high fence densities in critical wildlife areas such as pronghorn migratory pathways or sage grouse lekking habitat. Such novel fence data can assist wildlife and land managers to assess effects of anthropogenic features to wildlife at various scales; which in turn may help conserve declining grassland species and overall ecological functionality.

## Introduction

Worldwide, most long-distance terrestrial migrations have been lost [1] or greatly reduced [2] largely due to anthropogenic factors creating barriers to wildlife movements [1]. Restricted access to food and water is a primary threat to migrations worldwide [1]. In particular, fencing has played a large role in ungulate population declines where fencing closes off parks, delineates national boundaries and separates rangelands, thus physically cutting off access to necessary resources [3]–[8], [1] and in grassland bird decline, where birds may not be able to see fencing and die due to collision or entanglement [9]–[11]. In Africa, fences were implemented in the 1950's to prohibit transfer of wildlife disease between livestock and wildlife populations [1]. In Kruger National Park, fencing restricted migrations of wildebeest and the population, cut off from seasonal water sources, declined by nearly 88% [3]. In North America, 75% of seasonal migrations, mostly those of bison (*Bison bison*) and pronghorn antelope (*Antilocapra americana*) have been lost largely due to overhunting and disruption of migration routes [2] and it is hypothesized that fence collisions have contributed to the decline of sage grouse populations [12].

Wire fencing was introduced in the North American west by the homesteaders of the 19^th^ century to avoid importing timber or stone to create barriers around their land [13]. By the late 19^th^ century, barbed wire had been invented and was being produced commercially at the high demand of homesteaders wishing to claim their land [14]. By 1880–1884, barbed wire production reached a peak with an estimated 643,000–965,000 km being produced annually [13]. Eventually ranchers and farmers in the west began using fencing pervasively. Subsequently, access to roads and water holes were inadvertently restricted for cattle [14].

Today much of the fencing erected in the 1800's still exists and is profuse across northern Montana. It is used by land owners to delineate property, section off agricultural fields, demarcate parcel boundaries of the same ownership, corral cattle, and along roads for safety. Fencing type in northern Montana varies with land ownership and operation type. Three and four strand barbed wire are most common, however, mesh woven fences are also common in the Hi-Line region of northern Montana (A. Jakes, University of Calgary, *unpublished data*) and these wire fences can pose significant impediments to the movements of wildlife, thus modifying habitat availability and migration opportunities of various native species [1], [3], [7]. As such, fences can influence a particular species movement patterns at multiple spatial and temporal scales [15]. Species perceive the surrounding landscape differently from one another to fulfill life-history requirements [16], [17]. Appreciating that wildlife may have multi-scaled responses to anthropogenic factors such as fences requires that we too address anthropogenic factors at various scales.

Loss of migrations could result in extirpation of wildlife populations, resulting in overall contraction of species range due to decreased access to forage and safe calving grounds. Thus, to conserve migrations and allow continued movement to optimal seasonal ranges, conservation and maintenance of habitat outside of protected areas is required. Because wildlife generally track high quality forage and water sources through their migrations, erecting fencing along or through migration routes or breeding habitat can restrict access to necessary habitat, resulting in declines in population numbers [1], [18], [11]. For example, in a study in Colorado and Utah, USA, most mule deer (*Odocoileus hemionus*), elk (*Cervus canadensis*) and pronghorn (*Antilocapra americana*) fence-related mortalities were due to becoming entangled in fence wires [19]. Furthermore pronghorn density and fence-related mortality were positively correlated; indicating fencing may negatively impact pronghorn populations [19]. In addition, increased indirect mortalities of fawns being separated from does was observed from woven wire fences as opposed to plain barbed wire fences and additionally, mortalities increased with increasing fence height [19]. In a region where fencing is a common landscape feature such as northern Montana, migratory opportunities could be diminished and as a result, population numbers of wildlife could suffer declines unless landscape permeability is maintained [20].

Although North American migrations are among the best studied, and despite the importance of fencing information on wildlife movements at multiple scales, regional datasets on fence locations and fence densities do not exist. In this study we create a regional fence location and density model for the Hi-Line area of northern Montana. To aid in future studies identifying wildlife movement pathways or identifying factors affecting seasonal habitat selections we determined it was important to have a readily available predicted fence location and density information for this region. We provide methods for modeling fence locations and density at a large scale, using publicly available information in an effort to encourage the creation of fence spatial datasets for wildlife research and management in other regions.

## Methods

### Study Area

**Figure 1:**
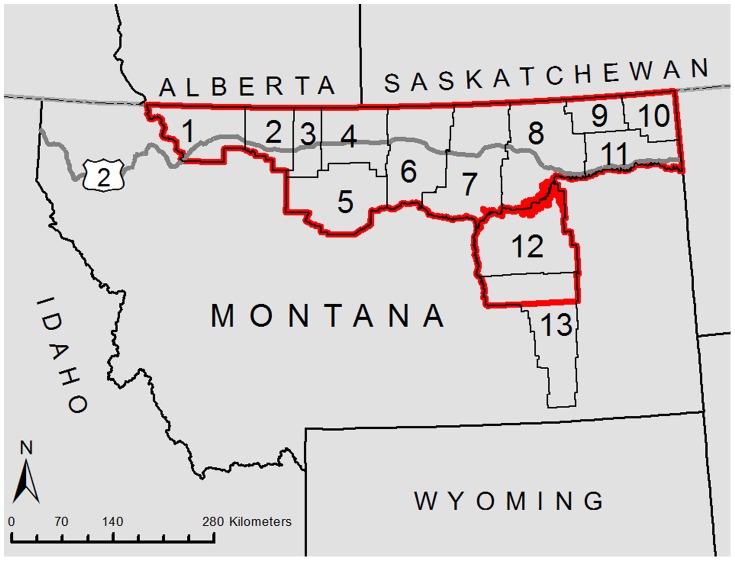
The location of the study area, the Hi-Line region of Montana (red), 2011. Counties included in the study are: 1 Glacier; 2 Toole; 3 Liberty; 4 Hill; 5 Chouteau; 6 Blaine; 7 Phillips; 8 Valley; 9 Daniels; 10 Sheridan; 11 Roosevelt; 12 Garfield; 13 Rosebud (northern half).

The study area is comprised of 13 counties in the Montana Hi-Line region of northern Montana (Figure 1). These counties are bounded by the Canadian border on the north and the Marias and Missouri Rivers to the south (Figure 1). The study area was chosen initially due to ongoing research on pronghorn migrations and movements at the northern terminus of pronghorn range (completed under wildlife capture and handling permit #11-2007 from the Montana Fish, Wildlife & Parks Institutional Animal Care and Use Committee). The total area for which fencing was modeled was 103,426 km^2^ (Table 1). Privately owned land is the dominate tenure type, more than double public land ownership (Table 1, Figure 1).

**Table 1:** Land ownership within the Hi-Line region.

Ownership	Area (km^2^)	% Study Area
Private	67879	65.63
US Bureau of Land Management	13736	13.28
Tribal	9401	9.09
State Government	7105	6.87
US Fish and Wildlife Service	2619	2.53
US National Park Service	1469	1.42
Water	416	0.40
USDA Forest Service	242	0.23
Local Government	213	0.21
US Bureau of Reclamation	205	0.20
Right of Way	85	0.08
US Department of Defense	26	0.03
US Department of Interior	19	0.02
Undetermined	7	0.01
US Government, Other	4	0.004
Total	103,426	

### Ground Truth Surveys

#### Transect Identification

A seamless land cover dataset developed by bordering state and provincial wildlife agencies was clipped to the bounds of the study area. From this land cover data, three generalized habitat type regions were delineated by extracting polygons around land cover of similar types. Habitat types included “grassland”, “agriculture” and “shrubland”. To create a fourth generalized habitat type, we used removed areas not previously defined and classified as “mixed” habitat to include the remaining areas. Next, we identified roads, paved or unpaved, from the roads dataset created by the Northern Sagebrush Steppe Initiative and intersected this layer with the generalized habitat type region within the Montana portion of the Northern Sagebrush Steppe Initiative land cover data. New areas were identified, based on both generalized habitat type and road type, leaving a total of eight different classifications: Grass/Unpaved; Grass/Paved; Agriculture/Unpaved; Agriculture/Paved; Shrub/Unpaved; Shrub/Paved; Mix/Unpaved and; Mix/Paved. Within each of these generalized areas, random points along roads were generated, each which served as the middle point of each 3.2 km road transect for field surveying. This distance was used to capture a majority of land tenure changes within the “checkerboard pattern” of land ownership found within this area. Random points were generated at a minimum distance of 3.5 km apart, to avoid transect overlap. Each random point had a unique transect associated with it and unique numbers were generated and manually entered for each random point. Transect identification took place in ArcGIS 9.3 (ESRI 2009) and random points and transect regions coordinates were defined in WGS 1984.

#### Field Surveys

**Figure 2:**
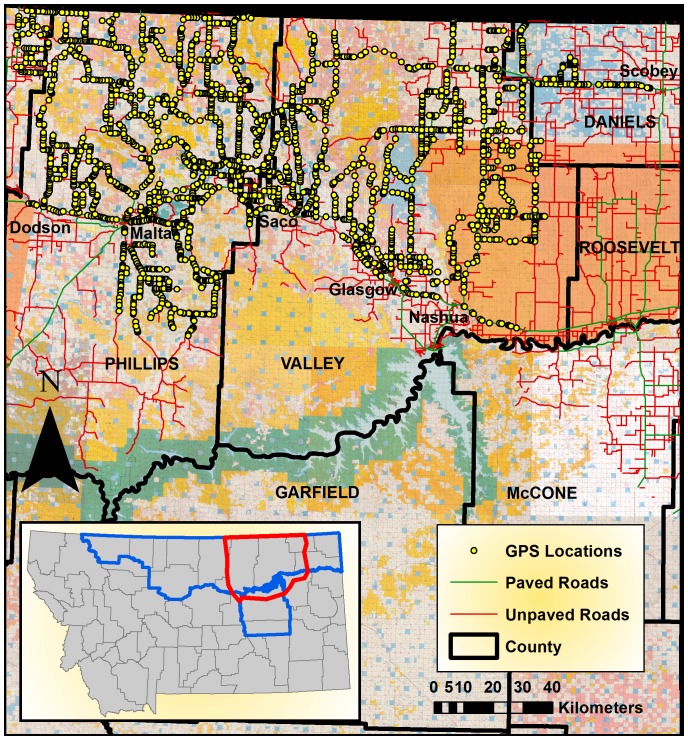
GPS locations of sampled fence transects during Summer 2009. Each GPS location represents a change in fence structure type or addition/deletion of fence along the sampled transect within four counties in northern Montana. Red outline – location of GPS sampling, Blue outline – the study area.

During June-August 2009, 2,362 km of fence along roadside transects were sampled in Blain, Phillips, Valley and Daniels Counties (Figure 2). In ArcGIS 9.3 (ESRI 2009), we created random midpoints for 3.2 km long survey transects. At each point along the 3.2 km transect that a roadside fencing ended, appeared, or changed in fence structure, a GPS location was recorded. Locations were also recorded at every interior fence and road intersection. Information on GPS locations, transect number and heading, fence structure, ground cover and road type was recorded. Locations and structure of internal fencing and twinning and tripled roadside fences were also recorded. Fencing had to be within 200 m of the road to be considered along the roadside and therefore within the transect. Additionally, changes in fence structure had to be longer than 100 m to be recorded. GPS waypoints were downloaded after each field day to create an overall layer of fence changes along roadsides or where interior fences converged with roadsides across the area. Complete sampling protocols (Text S1) were created to standardize methodologies for unique fencing schematics found across the landscape and are applicable to all survey areas across the study area.

### Fencing Location Modeling

Fence location and density were modeled using private land ownership data provided by the Montana Public Land Ownership dataset in ArcGIS 10.0 (ESRI 2012). We predicted fence presence based on parcel ownership, size and ownership adjacency. We used publicly available free datasets including land tenure data from the Montana Cadastral Database (Montana Department of Administration/Information Technology Services, 2011), pasture data provided by the Bureau of Land Management (Bureau of Land Management, 2011), the 2010 TIGER roads dataset (US Census Bureau, 2010) and land cover from the National Gap Analysis Program Land Cover Dataset (United States Geologic Service 2000) and the 2000 Land Cover for Agricultural Regions of Canada (Canada Agri-Geomatics Service 2000) national land cover dataset.

Historically, most ranches were based on ‘sections’ (2.59 km^2^) in this region of Montana, and we use this unit of measurement as our base unit of area. We began the modeling process with the land ownership polygon dataset. We edited this dataset based on a series of assumptions about where fencing exists around parcels, along roads and where it defines crop fields (Text S2). The remaining outlines of polygons after applying these assumptions and their associated GIS functions represented potential fencing. For example, we assumed private lands with the same mailing address <½ section in size are not fenced if adjacent to each other, so parcels <½ section that are privately owned are merged together in the GIS polygon dataset and the boundary of the two merged parcels is a fence. To create the assumptions, we consulted a variety of local experts, including BLM, Montana Fish, Wildlife and Parks, World Wildlife Fund, Montana Department of Natural Resources & Conservation, Rocky Boys Indian Reservation, Fort Belknap Indian Reservation and, Fort Peck Indian Reservation local personnel. Fence data for the Charles M. Russell National Wildlife Refuge (CMR) was provided, so this area was not included in the modeling efforts, but was added in for density estimates. We first modeled fencing by dissolving and merging the land tenure parcels based on the land tenure-based assumptions and then combined this dataset with the fencing modeled using the land cover-based assumptions. Within areas of large cropland, all land tenure-based fencing was removed, assuming there would be no parcel fencing within these areas, and fencing in these areas follows different rules (See below). Finally, roads-based fencing was overlaid on the two previously combined datasets.

#### Land Tenure Fence Modeling

To model fencing based on land ownership, we removed all BLM land from the land tenure layer and replaced it with pastures data we received from the BLM. We assumed the pasture polygon outline represented fences and that no additional fencing would exist on the BLM. Next, we eliminated the boundaries of neighboring state lands, assuming that if more than one state-owned parcel was adjacent, they would be fenced together. State-owned lands that were surrounded by BLM lands were dissolved into the BLM lands, assuming that the state lands would be leased to the BLM. Bureau of Reclamation lands were then treated the same as state lands, in that if two or more parcels were adjacent, they would be fenced together. This process was repeated to remove boundaries separating more than one Fish and Wildlife Service parcel. For private and tribal lands, we first merged parcels based on ownership and adjacency (parcels owned by the same party were combined as one if they were adjacent) and then selected resulting parcels that were greater than 2 sections (5.2 km^2^). These parcels were combined with the overall fence layer. The smaller parcels were dissolved into a neighboring parcel of similar size (5.2 km^2^) and then combined with the fence dataset. Next, we removed fencing on National Park Service and Forest Service land. This resulted in the final layer representing land tenure fencing.

#### Road Fence Modeling

We identified primary and secondary roads and buffered them using the estimates of road width as buffer width. These buffer outlines, 19 m wide on primary roads, and 11 m wide on secondary roads then approximated fence lines along roads. These square-ended buffers were dissolved, merged and converted to lines. So fences would not bisect roads, we removed the buffer ends by removing line segments that were exactly 38 m long, in the case of primary roads, and 22 m long in the case of secondary roads. This process left fence lines on either side of these roads. To model fencing along local roads, we first removed roads from the CMR. Next, length was calculated and long local roads (≥1,200 m) were merged with primary and secondary roads. Because local roads were assumed to have fencing only on one side, we used these local road polylines to represent fencing along them. To include local fenced roads in the model without including the misclassified local roads which were two-tracks or driveways we then selected short roads (<1200 m) that intersected long local roads. This began an iterative process where increasingly smaller roads which intersected larger roads were selected and added to the fenced roads classification. This process ensured small line fragments (two tracks, trails, and possible data errors) from the roads dataset were not included in the fence dataset. We merged the fenced short local roads identified through this process with longer local roads. We buffered this combined dataset by 11 m on each side, and converted the buffer to a line. One side of this double line was erased, and the remaining line represented fencing. Again, the remaining buffer ends were removed, and the resulting dataset of one-sided fencing along local roads was combined with the double-sided fencing along primary and secondary roads, defined by the US Census TIGER roads classification.

#### Land Cover Fence Modeling

The land cover layer was first converted to a polygon shapefile. To remove inconsistencies and small sections of non-cropland within large areas of crop (usually wheat and corn in this area) we identified crops, dissolve these polygons together and then calculated their area. We did the same for non-crop land cover classes. Next, we identified non-crop polygons ≤½ section (1.3 km^2^) that intersected large crop (≥3 sections or 7.8 km^2^). These non-crop sections were dissolved into the large areas of crop.

Based on advice we received from experts, we assumed there would always be a fence line between large areas of cropland (≥3 sections or 7.8 km^2^) and larger areas of native prairie (half of a section or >1.3 km^2^). These areas of prairie were identified and erased from the large sections of crop, which created a polygon boundary. This layer of large croplands was then converted to a raster, reclassified, and expanded from within to remove any remaining holes. This was then again converted to a polygon and represented fences around large croplands, and between large croplands and native prairie. We erased all land tenure fencing from the areas of large croplands, assuming the only other fencing on large croplands would be those along roads.

#### Fence Dataset Synthesis

To combine the three polygon fence layers, roads, land tenure and land cover, we first removed additional fence lines from the land cover layer from the BLM layer, again assuming the polygon outlines of the BLM pastures dataset would represent all of the fencing on this land type. We erased boundaries of large lakes and rivers polygons and lines that intersected study area boundary lines. The polygon layers of parcel fencing and land cover fencing were then combined and this layer was converted to a line dataset. Assuming that there would not be parcel boundary fence parallel to nearby roads fencing, we next removed roads fencing that were completely within a 20 m buffer of other types of fencing. Finally, the remaining roads fencing was merged with the land cover and land tenure fencing.

### Model Accuracy Assessment

Because fencing along roads was modeled separately from internal parcel fencing, we completed individual accuracy assessments for these portions of the fence model, as well as a combined accuracy assessment. For the roads accuracy assessment, all fenced transect lines (a fence on either or both sides) were merged and clipped to the accuracy assessment study area to identify the true positives and all non-fenced transects were merged, clipped, and used to identify the true negatives. These line transects were then converted to points and buffered by 30 m to account for spatial error. Only modeled fencing from the roads fencing model were used in this assessment. We identified areas where buffered GPS points representing true positives and true negatives intersected modeled fences along roads, to result in the true positives and false positives, respectively. The number of true positives was subtracted from the total number of fence transect points to result in the number of false negatives. The number of false positives was subtracted from the total number of non-fenced transect points to identify the number of true negatives. Total number of samples was 1,832 for true positives and 469 for true negatives.

For the internal fencing accuracy assessment, all GPS points were merged and clipped to the accuracy assessment study area to identify the true positives. We selected GPS locations that were along fenced transects and these points were then buffered by 30 m. We isolated the modeled internal fencing by removing roads fencing and identifying intersections in the modeled internal fencing. The points of intersection were then buffered by 30 m. To identify true positives, we identified where these buffered modeled intersection points intersected the buffered GPS points. The true positives were then subtracted from the total number of GPS points used to result in the false positives. To identify the true negatives, the buffered areas around the GPS sample points were subtracted from the fenced transects to ensure we used actual areas with fences, but with no internal fences (indicated by GPS locations) for this assessment. On the remaining transect lines, where fencing was not observed, we created random points and buffered them by 30 m. Identifying locations where these buffered points intersected modeled internal fences resulted in the false positives. The false positives were then subtracted from the total number of random points to result in the true negatives. Total number of samples was 1,333 for true positives and 1,290 for true negatives.

To calculate the total accuracy of the fence dataset, all GPS locations and the modeled fencing were buffered by 30 m to account for spatial error. Random points were created along the non-fenced transects and similarly buffered by 30 m. GPS locations intersecting modeled fencing resulted in the true positive rate and random points along non-fenced transects intersecting modeled fencing resulted in the false positive rate. The number of true positives were subtracted from the total number of GPS locations to result in the false negative rate and the number of false positives were subtracted from the number of non-fence transect random points to result in the true negatives. There were 1,655 samples for the true positive and the number of samples for true negative as 1,113.

We then calculated Cohen's Kappa, an accuracy measure commonly used in remote sensing applications [21]. The Kappa statistic is the chance agreement subtracted from the observed accuracy divided by chance agreement subtracted from 1. The Kappa statistic can range from −1 to 1, where 1 represents 100% accuracy, and 0 represents accuracy no better than that due to chance. Negative values are rare and generally indicate accuracy worse than random; a mismatch between ground truth locations and modeled data. Cohen's Kappa and confidence intervals were calculated in R statistical software (R Core Development Team 2012), using the FMSB package [22], which tests the null-hypothesis that the agreement between the model is the same as random, with Kappa  = 0.

Because the advice we received from our experts (local personnel from various organizations) about fencing on large croplands and BLM lands was variable we completed additional accuracy assessments. To identify causes of inaccuracies, we first removed BLM land from the model and repeated the accuracy assessment, and then removed land cover fencing.

### Fence Density

After we created the fence model, the density of fences was calculated using ArcGIS 10.0. The density function search radius for the entire study area was 10,000 m and cell size was 1500 m.

## Results

### Fence Location and Density Modeling

**Figure 3:**
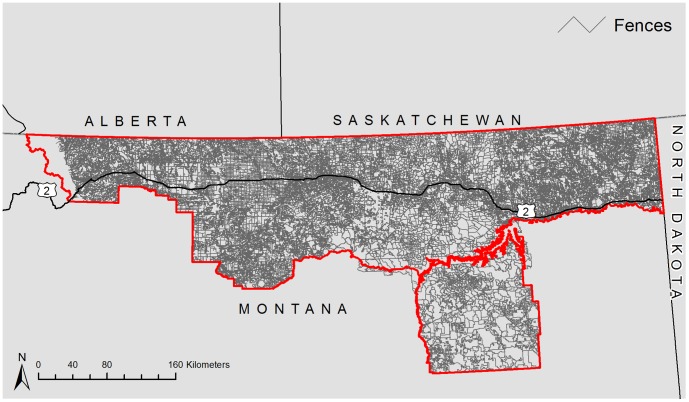
Modeled fences in the Montana Hi-Line region using land tenure, land cover and roads data.

**Figure 4:**
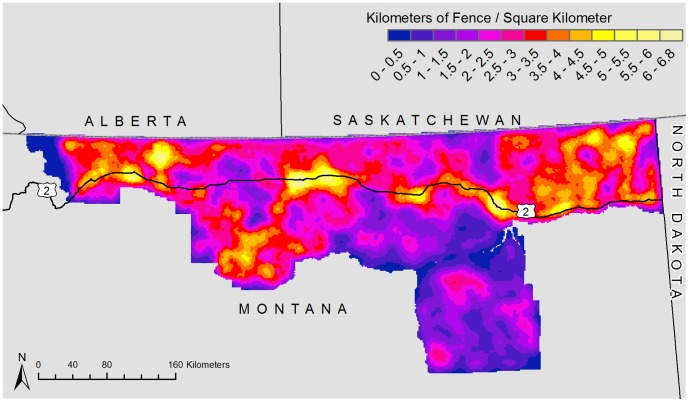
Fence density from modeled fence lines in the Montana Hi-Line region using publically available data.

A total of 263,308 km of fencing was predicted (Figure 3). Maximum fence density was 6.79 kilometers of fencing per km^2^ (Figure 4). Mean fence density was 2.37 km of fencing per km^2^. Fence density was highest along the U.S. Highway 2 corridor. Roosevelt County had the highest average density at 3.40 km/km^2^ and Rosebud County had the lowest density at 1.22 km/km^2^ (Table 2).

**Table 2:** Average fence density by county within the Hi-Line region.

County	Km Fence/Km^2^
Roosevelt	3.4
Sheridan	3.16
Toole	3.08
Daniels	2.97
Chouteau	2.81
Hill	2.74
Glacier	2.59
Blaine	2.55
Liberty	2.52
Valley	2.35
Phillips	2.06
Garfield	1.33
Rosebud	1.22

### Model Accuracy Assessment

To compare how well our different decision rules reflected true fence lines, we created three fence datasets: all fencing on the landscape; all fencing except fencing associated with BLM lands; and all fencing except fencing associated with large crop lands. From these three layers, we then calculated accuracy for all fence types together; fencing only along roads and fencing only around land parcels. We chose to examine the rules associated with BLM lands and large croplands because our experts suggested these rules may vary greatly across the landscape.

Accuracy for all fencing and all types of fencing within the dataset was more accurate than random (Kappa  = 0), with a Kappa of 0.40 (Table 3). Within this dataset, the accuracy of the roads was lowest with a total accuracy (proportion of ground truth points that matched modeled fencing) of 0.63 and Kappa of 0.12.

**Table 3:** Accuracy assessment results for the complete fence layer.

	Total Accuracy	Kappa	Confidence Intervals*
Roads	0.63	0.12	0.07–0.17
Internal	0.64	0.29	0.26–0.33
Total	0.73	0.40	0.36–0.44

*95% confidence intervals.

In the dataset excluding fences associated with BLM land, we found a consistent decrease in accuracy (Table 4). When assessing the accuracy of the roads fencing only, Kappa was −0.07. The internal (parcel) fencing was the most accurate within this layer, with a Kappa of 0.28.

**Table 4:** Accuracy assessment results for the fence layer without BLM land.

	Total Accuracy	Kappa	Confidence Intervals*
Roads	0.65	−0.07	−0.14–−0.01
Internal	0.63	0.28	.24–.31
Total	0.60	0.18	0.13–0.22

*95% confidence intervals.

Accuracy was highest when large areas of cropland were excluded, in the third fence dataset. In this assessment, Kappa was 0.56. In analyzing accuracy for roads fencing in this dataset, Kappa was 0.27 and Kappa for land tenure fencing was 0.41 in the absence of fencing associated with large croplands (Table5).

**Table 5:** Accuracy assessment results for the fence layer without large croplands.

	Total Accuracy	Kappa	Confidence Intervals*
Roads	0.72	0.27	0.22–0.33
Internal	0.72	0.41	0.37–0.45
Total	0.78	0.56	0.53–0.60

*95% confidence intervals.

## Discussion

Although these fence location and density datasets can benefit from improvements in data analysis and sampling methods, this exercise did produce noteworthy results. Higher fence densities appear along Highway 2, where residential areas contributed to the increase in density. Less developed areas, such as the CMR and Glacier National Park contribute to the areas of low fence density (Figure 4). From our accuracy assessment, our model displayed actual fence locations along predicted fence lines at a Kappa of 0.40–0.56, considered moderate agreement between the modeled and ground truth data [23]. Therefore, using this modeling approach offers moderately accurate fence locations and density over a large spatial scale. In addition, regional rules can be created to hone the methodology to specific states or provinces of interest.

Our accuracy varied slightly depending on whether croplands and BLM lands were included in the model. Because these decision rules were based on expert opinion and experience, and our experts were not as confident with large areas of cropland and BLM lands, we believe that adding experts from these areas may further improve model accuracy. In some areas of the West, fences are used on BLM lands. Since we assumed the BLM pasture boundaries were fences on BLM land, accuracy may be increased when other fence types are included in the model. We also assumed that fencing on areas of croplands would be different than non-cropland areas, however, the increase in accuracy gained when removing the croplands from the model may suggest otherwise. Accuracy was lowest when fencing associated with large croplands were included in the fence dataset, but we believe that our model overall is an accurate reflection of fencing on the landscape.

Improvements in our analysis methods may improve our overall accuracy of the fence dataset. Different fence structure types have different effects on wildlife movement and habitat selection [19] and the fence layer created here did not include fence structure data. Fence structure type is difficult to model over large regions and is more a result of private landowner's preference and type of livestock production per ranch. Wire mesh fencing has been used in this study area to corral sheep and may be particularly hard for wildlife to cross. Additional improvements in accuracy of the fence layer may be made by amending the fence sampling protocol. Fence surveyors at times recorded fence locations at as much as 100 m from the actual fence due to railroad right-of-ways and property rights and interior pasture fences off roadways were not ground-truthed. Future sampling forays are planned to sample more of the study area, which will be imperative for future landscape fence permeability analyses for wildlife. Finally, because this data was created with the continued reliance on assumptions about neighboring parcels of land, there may be a decrease in accuracy around the edges of the study area. We recognize that individual fences may not be accurately modeled. Although improvements may be made, this novel effort uses extractable methodologies we believe will assist in modeling a key variable towards unraveling wildlife movement and habitat selection.

Fences can exhibit both indirect and direct effects on native wildlife populations worldwide [24]–[26], [11], [10]. In North America, indirect effects of fencing such as animal displacement, reduced habitat availability, and habitat fragmentation may have a higher impact on pronghorn and other wildlife populations than direct effects, by altering behavior and movement rates resulting in eventual population decline [], [1], [19], [25], [27,28]. Concerning pronghorn, and in particular during sever winter conditions, snow and ice can accumulate over the bottom-most fence wire during winter, thus preventing pronghorn from crawling underneath fences. Because of this, fencing may prevent migrating pronghorn from reaching higher quality habitat, during which time they have expended energy without finding better conditions. Fencing therefore can alter behavior at multiple scales and place North American wildlife in perilous situations as it has in other areas of the world [3], [1], [10], [11], [18].

A regional fence layer allows both wildlife and land managers to assess effects to wildlife at various scales, including at home range and within home range level of habitat selection [15], as well as identifying important population-level movement pathways between seasonal ranges during migration. Certainly, it can aid researchers through inclusion into predictive modeling efforts to assess habitat suitability and connectivity at regional level scales. As a priority, on-the-ground management practices could identify high fence densities (here along the developed Highway 2 (Figure 4)), along migratory pathways and within breeding grounds; ecological necessities to sustain North America's dwindling grassland wildlife [29]. Federal, state and provincial agencies, along with non-profit organizations and community organizations all can play an important role by undertaking cooperative projects to modify fences in strategic locations. These could include key-linkage areas which are geographic or anthropogenic areas and/or critical stopover locations along the migration pathway and fawning and/or lek areas. Without planning and the proper data, the cumulative anthropogenic changes to landscapes will continue to erode wildlife habitats and seasonal migration opportunities, reducing effective habitat patch size, potentially leading to sustained population declines and contraction of overall species range.

## Supporting Information

Text S1 **Field Fence Surveying Protocol.**
(DOCX)

Text S2 **Assumptions used to model fencing.**
(DOCX)
