# Supplementary material for: Modeling Fence Location and Density at a Regional Scale for Use in Wildlife Management
Source: PLoS One. 2014 Jan 8;9(1):e83912. doi: 10.1371/journal.pone.0083912 (PMC3885515; doi:10.1371/journal.pone.0083912)
Supplement: Text S1 — Field Fence Surveying Protocol. (DOCX) [file pone.0083912.s001.docx]

Text S1. Field Fence Surveying Protocol

Recording Data in Excel Spreadsheet:

- Excel Spreadsheet column headings: Date, Transect, Waypoint, Heading, Road Class, Land cover (General), Land cover (Ground), Roadside Fencing (Driver side), Heading of Driver Side Fencing, Type of Driver Side Fencing, Roadside Fencing (Passenger Side Fencing), Heading of Passenger Side Fencing, Type of Driver Side Fencing, Interior Fencing Present, Interior Fencing Direction, Interior Fencing Type, Length of Transect, Structures Present, Notes.
- First row in Excel spreadsheet depicts fence presence/absence and structure type at the start of random transect “X” (1.6 km away from random point) and receives a GPS waypoint; coded as “XXXXA”. An additional row in the spreadsheet is added for each transect where there is a change in roadside fencing or where interior fencing exists. Examples include where an additional fence parallels the road, where there is a fence corner, where a fence changes structure type.
- The “Heading” provides direction of transect from start to finish. For example, driving South to North would be written S-N; if direction changes during the transect such as while turning corners, then the heading changes.
- “Land cover (General)” is the land cover type identified by GIS layer.
- “Land cover (Ground)” is land cover type identified by surveyor along transect. For example, if “Land cover (General)” is identified as “native” and the area is actually identified as “agriculture”, then record as “agriculture”.
- Where two interior fences come from the same heading, one fence is recorded as “Interior Fencing”, the second is recorded in the “Notes” column as “Second heading fence is “X” strand barbed-wire and permeability code “X”.
- In “Notes”, “dbl = 0” identifies that a double driver side/passenger side or paralleling fence has stopped.
- Where no fencing is present along transect, this transect should have one row with one beginning waypoint only.
- In situations where the middle point (random point) of transect is moved, record the new GPS waypoint following “Recording GPS Waypoints” guidelines below; no rows in Excel spreadsheet are added.

Transect Sampling Rules:

- Recordable roadside fencing has to be within ~100 m of road (close enough that surveyor can code the permeability).
- Recordable fence segment needs to be at least 200 m long if an interior fence, 100 m long if fence parallels the road.
- A fence is never double counted at various stops along the transect; only record if there is an addition, deletion or structure change to the fence.
- “Driver Side Fencing” is fencing located to the driver’s left; “Passenger Side Fencing” is always to driver’s right.
- Fencing around structures (farm houses, corrals, etc.) is not recorded but structures are recorded in “Structure” column.
- Where fencing parallels the road from a distance further than 100 m changes from one structure type to another (or changes from absent to present or present to absent), the change is recorded in new Excel row. However, often it is impossible to observe when a parallel fence comes in or goes away. As a result, waypoints are not recorded for parallel fences.
- When classifying the heading of a “double fence” further than 100 m that parallels the road: Classify based on the direction of transect heading and lane.
  - Example: Traveling South to North, heading would be “North”. A double fence starts that parallels the road; heading would be “North” if fence was off to the right-hand side (vehicle is in right-hand lane) and receive a “South” heading if fence was off to the left-hand side.
- If a fence is observed on the far side of a railroad which parallels the road, that fence is recorded as a “parallel fence”, not as “driver side” or “passenger side” fence. Consequently, the fence does not receive a waypoint.
- On two-track roads, and going through a gate (perceived as discontinuance of fence), record the fence as being two interior or perpendicular fences.

Recording GPS Waypoints:

- Transect waypoints are named as the transect number and next letter in alphabet. For example, begin as 7641A and the next fence change is recorded as 7641B, etc.
- Where transect is moved to fit 3.2 km distance, the middle waypoint receives that transect number only (no letter). In these instances, use the vehicle odometer to record the distance to middle point and 1.6 km past the middle point to the end of transect.
- Each transect is treated as separate. As such, when crossing an intersection with a previously sampled transect, record data from the present transect independently.
- Do not record GPS waypoints for “double fences” that parallel the road, but the surveyor should record it in a new Excel row when one of the fences changes structure type.

Fence Permeability Codes:

- Code 1: ≥ 16 in. lowest barbed-wire strand;
- Code 2: Animal may travel ~ 100 m. to find gap ≥ 16 in. lowest barbed-wire strand;
- Code 3: Animal may travel ~100-300 m. to find gap ≥ 16 in. lowest barbed-wire strand;
- Code 4: Challenging to cross except for topographic dips, typically < 16 in. lowest barbed-wire strand;
- Code 5: Impermeable barbed-wire fence typically ≤ 12 in. lowest barbed-wire strand;
- Code 6: Woven wire or picket fence.

Selecting Subjective Fence Permeability Codes:

- When selecting between Code 1 and Code 2 for a three-strand barbed wire fence, always select “Code 1” if it is 16 in. off ground, even if it’s sagging between fence posts.
- Three-strand barbed wire fence typically receives a highest permeability code of “Code 3”. The only instances these fences receive a Code 4 is if it’s a well-constructed new fence and the lowest strand is ≤ 12 in. lowest barbed-wire strand.
