# Supplementary material for: Modeling Fence Location and Density at a Regional Scale for Use in Wildlife Management
Source: PLoS One. 2014 Jan 8;9(1):e83912. doi: 10.1371/journal.pone.0083912 (PMC3885515; doi:10.1371/journal.pone.0083912)
Supplement: Text S2 — Assumptions used to model fencing. (DOCX) [file pone.0083912.s002.docx]

Text S2. Assumptions used to model fencing

Land Tenure-based Assumptions:

- Private lands with the same mailing address < ½ section are not fenced if adjacent to each other.
- Private lands with the same mailing address are not fenced if, when adjacent, they are > 2 sections.
- Publicly-owned parcels >5 sections of one ownership type are fenced if adjacent to similarly large parcels of publicly owned lands of another ownership type.
- State-owned parcels are not fenced if they are within US FWS lands or within BLM pasture lands.
- The boundary of state-owned parcels is fenced if it is adjacent to (but not within) BLM lands.
- Bureau of Reclamation-owned parcels sharing a border are not fenced.
- Parcels ≤1/16^th^ of a section of any ownership type are fenced within urban areas. Along the Milk River, these areas would not be fenced.

Land cover-based Assumptions:

- The boundary between cultivated crop fields ≥ 3 sections and native prairie are fenced regardless of private land ownership. If cultivated crops overlap public land, the fencing follows the land ownership assumptions
- Large contiguous patches of cultivated crop fields ≥ 3 sections are not fenced regardless of ownership. Within these large patches of cultivate crops, the border of native prairie parcels of ≥ ½ of a section are fenced.

Roads-based Assumptions:

- There are no fenced roads on the Charles M. Russell National Wildlife Refuge except major highways.
- Primary and secondary roads (as defined by TIGER) are fenced on both sides regardless of land ownership. From field estimates, fences were place on 19m from either side of primary and secondary roads.
- Local roads are fenced on one side regardless of land ownership.
- Four wheel drive and two track roads are not fenced.
- If roads fencing falls within 5m of a fenced parcel boundary, the roads fencing is removed, to eliminate redundant fencing.
